# *Vibrio cholerae* Non-O1, Non-O139 Serogroups and Cholera-like Diarrhea, Kolkata, India

**DOI:** 10.3201/eid1903.121156

**Published:** 2013-03

**Authors:** Devarati Dutta, Goutam Chowdhury, Gururaja P. Pazhani, Sucharita Guin, Sanjucta Dutta, Santanu Ghosh, K. Rajendran, Ranjan K. Nandy, Asish K. Mukhopadhyay, Mihir K. Bhattacharya, Utpala Mitra, Yoshifumi Takeda, G. Balakrish Nair, Thandavarayan Ramamurthy

**Affiliations:** Author affiliations: National Institute of Cholera and Enteric Diseases, Kolkata, India (D. Dutta, G. Chowdhury, G. P. Pazhani, S. Guin, S. Dutta, S. Ghosh, K. Rajendran, R.K. Nandy, A.K. Mukhopadhyay, M.K. Bhattacharya, U. Mitra, Y. Takeda, T. Ramamurthy);; Translational Health Science and Technology Institute, Haryana, India (G.B. Nair)

**Keywords:** Vibrio cholerae, bacteria, non-O1, non-O139 serogroups, nonagglutinating vibrios, NAG, enteric infections, cholera-like diarrhea, virulence genes, pulsed-field gel electrophoresis, India

## Abstract

We identified 281 *Vibrio cholerae* non-O1, non-O139 strains from patients with diarrhea in Kolkata, India. Cholera-like diarrhea was the major symptom (66.0%); some patients (20.3%) had severe dehydration. These strains lacked the *ctxA* gene but many had *hlyA*, *rtxA*, and *rtxC *genes. Pulsed-field gel electrophoresis showed no genetic link among strains.

*Vibrio cholerae* O1 has been responsible for several cholera outbreaks in developing countries. During 1992, a novel serogroup, O139, caused cholera outbreaks in India and other countries in Asia (*1*). These events have shown that serogroups other than O1 have major epidemiologic roles in cholera. *V. cholerae* O1 and O139 serogroups produce cholera toxin (CT), a critical virulence factor and express toxin coregulated pilus (TCP), which are responsible for secretory diarrhea and intestinal colonization, respectively. Serogroups other than O1 and O139 are designated as *V*. *cholerae* non-O1, non-O139, or nonagglutinating vibrios (NAGs); such serogroups have >200 somatic (O) antigens (*2*) and mostly lack CT- and TCP-coding genes.

Toxigenic and nontoxigenic NAGs have caused several diarrhea outbreaks in India and other countries, including Haiti (*3**–**6*). In non–CT-producing NAGs, other virulence factors such as heat-stable enterotoxin (Stn), hemolysin (HlyA), repeat in toxin (RTX), and type 3 secretion systems (TTSS) have major roles in causing infections (*7*). In this study, we analyzed clinical characteristics of hospitalized patients with diarrhea infected with NAGs and screened strains for antimicrobial drug susceptibility, virulence genes, and genetic relatedness.

## The Study

During 2002–2010, a total of 12,719 fecal specimens were collected, which represented every fifth hospitalized diarrhea patient at the Infectious Diseases Hospital in Kolkata and all children at the outpatient unit at B.C. Roy Memorial Hospital for Children in Kolkata. Fecal specimens were screened for *V. cholerae* and other enteric pathogens as described (*8*). NAGs were serotyped by using 206 polyclonal O antisera according to the protocol developed at the National Institute of Infectious Diseases (Tokyo, Japan) (*2*).

Antimicrobial drug susceptibility assays were performed by using the disk diffusion method and commercially available disks (Becton Dickinson, Sparks Glencoe, MD, USA), according to standards of the Clinical and Laboratory Standards Institute (*9*). Because these standards do not include interpretive criteria for *V. cholerae*, breakpoints for *Enterobacteriaceae* were adopted. *Escherichia coli* ATCC 25922 was used as a quality-control strain.

Simplex and multiplex PCRs were performed by using published methods specific for *ctxA*, *tcpA*, *rtxA*, *rtxC*, *stn*, and *hlyA* genes (classical/El Tor) and the TTSS-coding genes (*7*). Pulsed-field gel electrophoresis was performed according to the PulseNet standardized protocol for *V. cholerae* (www.pulsenetinternational.org/SiteCollectionDocuments/pfge/5.71_2009_PNetStandProtVcholerae.pdf. Gel Compare II software (Applied Maths NV, Sint-Martens-Latem, Belgium) was used for comparison of electrophoresis patterns. This software uses a Dice similarity index and contains an unweighted pair group with arithmetic mean method.

Of the 12,719 diarrhea feces specimens screened, 2,206 (17.3%) contained *V. cholerae*, which included *V. cholerae* O1 in 1,841 (83.4%), and O139 in 84 (3.8%). In the remaining 281 (12.7%) specimens, *V. cholerae* strains did not agglutinate with serogroups O1 or O139. This result was confirmed by species-specific *ompW* PCR, which included strains collected during 2003 (*7*). Among 281 strains, 175 (62.3%) NAGs were the only enteric pathogen found, and 106 (37.7%) of those NAGs were found with other enteric pathogens. The isolation frequency of NAGs ranged from 1.2% to 3.2% (Table 1).

**Table 1:** Prevalence rates of *Vibrio cholerae* non-O1, non-O139 strains among patients with diarrhea, Kolkata, India

Year	No. fecal specimens	No. (%) serogroups isolated	Infection status	
			Single	Mixed
2002	2,285	49 (2.1)	30	19
2003	1,673	53 (3.2)	29	24
2004	2,430	31 (1.3)	16	15
2005	1,472	38 (2.6)	27	11
2006	930	19 (2.0)	12	7
2007	744	24 (3.2)	23	1
2008	1,124	35 (3.1)	22	13
2009	1,380	17 (1.2)	8	9
2010	681	15 (2.2)	8	7
Total	12,719	281 (2.2)	175	106

Although *V. cholerae* O1 is highly prevalent in Kolkata, more NAGs were detected than in our previous study (*10*). A total of 224 (79.7%) strains were categorized into 80 serogroups; the remaining 57 (20.3%) were untypeable. Among typeable serogroups, 14 (6.2%) strains belonged to the O37 serogroup, and 11 (4.9%) each belonged to serogroups O6 and O34. Serogroups O97 (4.5%), O11 (3.6%), and O59 (3.1%) were also identified in this study. Although serogroups O11, O35, and O37 showed a lower prevalence, their prevalence was higher among patients with diarrhea in Kolkata (*7**,**10*). The prevalence rate of NAGs (≈2%) in Kolkata is similar to that in the Haizhu District of Guangzhou, China (*11*). However, the number of serogroups identified in China (26 serogroups) was less than in our study (80 serogroups).

Among age groups of patients, NAGs were detected mostly in patients >5 years of age (>72%) than in those <5 years of age (≈28%) (Table 2). NAGs produce a spectrum of gastrointestinal symptoms ranging from asymptomatic infection to severe cholera-like illness or bloody diarrhea. In this study, most (70%) patients had watery diarrhea, which was similar to that for patients with cholera and those exclusively infected by NAGs (Table 2). Other clinical symptoms, such as dehydration status (22.3%) and fever (41.1%), were also high in patients with a single infection. In patients with mixed infections, bloody diarrhea (23.6%) and abdominal pain (41.5%) were the 2 major symptoms (Table 2), perhaps because >1 pathogen was involved.

**Table 2:** Clinical characteristics of *Vibrio cholerae* non-O1, non-O139 strain–infected patients with diarrhea, Kolkata, India

Characteristic	Infection status, no. (%)	
	Single, n = 175	Mixed, n = 106
Type of diarrhea		
Watery	122 (70.0)	63 (59.4)
Loose with bloody mucus	30 (17.1)	25 (23.6)
Other	23 (13.1)	18 (17.0)
Dehydration status		
Severe	39 (22.3)	18 (17.0)
Moderate	136 (77.7)	88 (83.0)
Age, y		
>5	126 (72.0)	75 (70.8)
≤5	49 (28.0)	31 (29.2)
Sex		
M	106 (60.6)	64 (60.4)
F	69 (39.4)	42 (39.6)
Fever		
Yes	72 (41.1)	39 (36.8)
No	103 (58.9)	67 (63.2)
Abdominal pain		
Yes	69 (39.4)	44 (41.5)
No	106 (60.6)	62 (58.5)

Most NAGs were resistant to nalidixic acid (57.6%), ampicillin (55.5%), furazolidone (36.6%), and streptomycin (32.4%) and highly susceptible to gentamicin (96%), tetracycline (80%), and chloramphenicol (80.4%). During 1992–1997, antimicrobial drug resistance was high among *V. cholerae* isolated in Kolkata (*12*). In this study, patterns of antimicrobial drug resistance in NAGs were different than those in previous reports (*10**,**12*). This finding might be caused by discontinuation of ineffective antimicrobial drugs, such as co-trimoxazole and furazolidone, rational use of fluoroquinolones, and introduction of azithromycin for treating diarrhea.

## Conclusions

Unlike our previous study (*7*), in this study, factors involved in the virulence of NAGs were not comprehensively elucidated in the study region. Unlike *V. cholerae* O1/O139, the pathogenicity of NAGs has been associated with >1 virulence factor (*7**,**10*). All NAGs lacked *ctxA* and El Tor *tcpA *genes. However, 6 (2.1%) strains had the *tcpA *gene; in 5 of them it was the only virulence gene detected. Most (94%) strains had the gene encoding El Tor type hemolysin, followed by *rtxA* (91.4%) and *rtxC* (75%) genes; only 5 (1.8%) had the *stn *gene. The RTX family includes a group of protein toxins produced by gram-negative bacteria, including *V. cholerae* with hemolytic, leukotoxic, and actin cross-linking activities, which may play a role in virulence (*13*). In this study, prevalence of NAGs that have genes encoding TTSS as the only virulence factor (41 cases) was higher than in Bangladesh and Argentina (*14**,**15*).

Strains containing *hlyA*, *rtxA*, and *rtxC* genes were predominant, followed by strains containing *hlyA-rtxA-rtxC*-TTSS and *hlyA-rtxA* genes (Table 3). Some gene combinations, such as *hlyA-rtxA-rtxC*-TTSS, *hlyA-rtxA-rtxC*, and *hlyA-rtxA*, were detected predominantly NAGs as the only virulence factor genes (Table 3). However, there was no correlation between type of serogroup and prevalence of putative virulence gene(s). Pulsed-field electrophoresis profiles of 70 strains representing the predominant serogroups showed distinct patterns (overall similarity ≈70%) (Figure). There was no profile match among strains belonging to the same serogroup.

**Table 3:** Distribution of virulence genes among *Vibrio cholerae* non-O1, non-O139 strains, Kolkata, India*

Gene	Infection status	
	Single	Mixed
*hlyA-tcpA(cl)-rtxA-rtxC*-TTSS	3	1
*hlyA-tcpA(cl)-rtxA-rtxC*	2	0
*hlyA-stn-rtxA-rtxC*-TTSS	3	2
*hlyA-rtxA-rtxC*-TTSS	40	29
*hlyA-rtxA-rtxC*	78	48
*hlyA-rtxA*	31	13
*hlyA-*TTSS	2	0
*rtxA-*TTSS	1	0
*rtxA-rtxC*	2	3
*hlyA*	9	3
*rtxA*	1	0
None	3	7

*hlyA, hemolysin A; *tcpA*, toxin coregulated pilus A; *rtxA, *repeat in toxin A; *rtxC*, repeat in toxin C; TTSS, type 3 secretion systems; *stn*, heat-stable enterotoxin.

**Figure:**
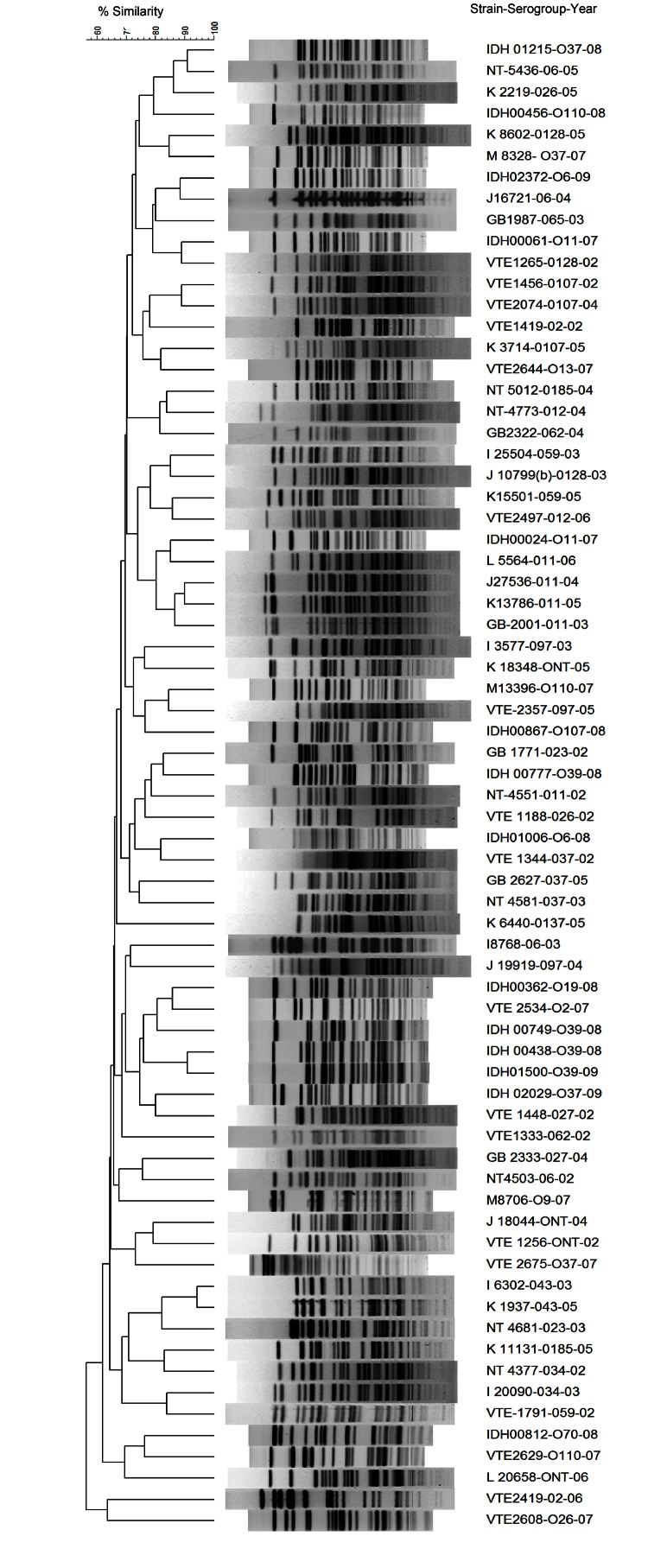
*Not*I restriction patterns of genomic DNA of representative *Vibrio cholerae* non-O1, non-O139 strains, Kolkata, India. Dendrogram was generated by using the unweighted pair group with arithmetic mean method.

Prevalence of NAGs associated with severe traits of infection indicates the role of these pathogens in cholera. The pathogenic mechanism of NAGs is multifarious; there are several virulence factors in genetically distinct strains. On the basis of our results, current antimicrobial drug therapy in the clinical management of NAG-mediated diarrhea can be continued. Further epidemiologic studies are needed to determine the ecology, virulence factors, and public health role of NAGs.

## References

[1] Ramamurthy T, Yamasaki S, Takeda Y, Nair GB. *Vibrio cholerae* O139 Bengal: odyssey of a fortuitous variant. Microbes Infect. 2003;5:329–44 . 10.1016/S1286-4579(03)00035-2 12706446

[2] Yamai S, Okitsu T, Shimada T, Katsube Y. Distribution of serogroups of *Vibrio cholerae* non-O1 non-O139 with specific reference to their ability to produce cholera toxin, and addition of novel serogroups [in Japanese]. Kansenshogaku Zasshi. 1997;71:1037–45. 9394556 10.11150/kansenshogakuzasshi1970.71.1037

[3] Dalsgaard A, Forslund A, Bodhidatta L, Serichantalergs O, Pitarangsi C, Pang L, A high proportion of *Vibrio cholerae* strains isolated from children with diarrhea in Bangkok, Thailand are multiple antibiotic resistant and belong to heterogenous non-O1, non-O139 O-serotypes. Epidemiol Infect. 1999;122:217–26. 10.1017/S0950268899002137 10355785 PMC2809609

[4] Rudra S, Mahajan R, Mathur M, Kathuria K, Talwar V. Cluster of cases of clinical cholera due to *Vibrio cholerae* O10 in east Delhi. Indian J Med Res. 1996;103:71–3 . 8714141

[5] Onifade TJ, Hutchinson R, Van Zile K, Bodager D, Baker R, Blackmore C. Toxin producing *Vibrio cholerae* O75 outbreak, United States, March to April 2011. Euro Surveill. 2011;16:19870 . 21616048

[6] Hasan NA, Choi SY, Eppinger M, Clark PW, Chen A, Alam M, Genomic diversity of 2010 Haitian cholera outbreak strains. Proc Natl Acad Sci U S A. 2012;109:E2010–7. 10.1073/pnas.1207359109 22711841 PMC3406840

[7] Chatterjee S, Ghosh K, Raychoudhuri A, Chowdhury G, Bhattacharya MK, Mukhopadhyay AK, Incidence, virulence factors, and clonality among clinical strains of non-O1, non-O139 *Vibrio cholerae* isolates from hospitalized diarrheal patients in Kolkata, India. J Clin Microbiol. 2009;47:1087–95 . 10.1128/JCM.02026-08 19158257 PMC2668327

[8] Nair GB, Ramamurthy T, Bhattacharya MK, Krishnan T, Ganguly S, Saha DR, Emerging trends in the etiology of enteric pathogens as evidenced from an active surveillance of hospitalized diarrhoeal patients in Kolkata, India. Gut Pathog. 2010;2:4. 10.1186/1757-4749-2-4 PMC2901208 20525383

[9] Clinical and Laboratory Standards Institute. Performance standards for antimicrobial susceptibility testing. Document M100–S21. Wayne (PA): The Institute; 2011.

[10] Ramamurthy T, Bag PK, Pal A, Bhattacharya SK, Bhattacharya MK, Shimada T, Virulence patterns of *Vibrio cholerae* non-O1 strains isolated from hospitalized patients with acute diarrhoea in Calcutta, India. J Med Microbiol. 1993;39:310–7. 10.1099/00222615-39-4-310 8411093

[11] Xu SH, Li YX, Li ST, Wu Q, Sun FQ, Huang F, Epidemic condition and biological characteristics of non-O1/non-O139 *Vibrio cholerae* in Haizhu District of Guangzhou [in Chinese]. Zhonghua Yu Fang Yi Xue Za Zhi. 2010;44:1087–90. 21215109

[12] Garg P, Chakraborty S, Basu I, Datta S, Rajendran K, Bhattacharya T, Expanding multiple antibiotic resistance among clinical strains of *Vibrio cholerae* isolated from 1992–7 in Calcutta, India. Epidemiol Infect. 2000;124:393–9. 10.1017/S0950268899003957 10982062 PMC2810924

[13] Olivier V, Salzman NH, Satchell KJ. Prolonged colonization of mice by *Vibrio cholerae* El Tor O1 depends on accessory toxins. Infect Immun. 2007;75:5043–51. 10.1128/IAI.00508-07 17698571 PMC2044531

[14] Rahman MH, Biswas K, Hossain MA, Sack RB, Mekalanos JJ, Faruque SM. Distribution of genes for virulence and ecological fitness among diverse *Vibrio cholerae* population in a cholera endemic area: tracking the evolution of pathogenic strains. DNA Cell Biol. 2008;27:347–55. 10.1089/dna.2008.0737 18462070 PMC2980768

[15] González Fraga S, Villagra de Trejo A, Pichel M, Figueroa S, Merletti G, Caffer MI, Characterization of *Vibrio cholerae* non-O1 and non-O139 isolates associated with diarrhea [in Spanish]. Rev Argent Microbiol. 2009;41:11–9 . 19391519

